# Diagnostic accuracy research in glaucoma is still incompletely reported: An application of Standards for Reporting of Diagnostic Accuracy Studies (STARD) 2015

**DOI:** 10.1371/journal.pone.0189716

**Published:** 2017-12-14

**Authors:** Manuele Michelessi, Ersilia Lucenteforte, Alba Miele, Francesco Oddone, Giada Crescioli, Valeria Fameli, Daniël A. Korevaar, Gianni Virgili

**Affiliations:** 1 IRCSS—Fondazione G.B. Bietti, Rome, Italy; 2 Department of Translational Surgery and Medicine, University of Florence, Florence, Italy; 3 Neurosciences, Psychology, Drug Research and Child Health (NEUROFARBA), University of Florence, Florence, Italy; 4 Ophthalmology unit, Department of Sens, Organs, University of Rome “Sapienza”, Rome, Italy; 5 Department of Clinical Epidemiology, Biostatistics and Bioinformatics (KEBB), Academic Medical Centre (AMC), University of Amsterdam (UvA), Amsterdam, The Netherlands; TNO, NETHERLANDS

## Abstract

**Background:**

Research has shown a modest adherence of diagnostic test accuracy (DTA) studies in glaucoma to the Standards for Reporting of Diagnostic Accuracy Studies (STARD). We have applied the updated 30-item STARD 2015 checklist to a set of studies included in a Cochrane DTA systematic review of imaging tools for diagnosing manifest glaucoma.

**Methods:**

Three pairs of reviewers, including one senior reviewer who assessed all studies, independently checked the adherence of each study to STARD 2015. Adherence was analyzed on an individual-item basis. Logistic regression was used to evaluate the effect of publication year and impact factor on adherence.

**Results:**

We included 106 DTA studies, published between 2003–2014 in journals with a median impact factor of 2.6. Overall adherence was 54.1% for 3,286 individual rating across 31 items, with a mean of 16.8 (SD: 3.1; range 8–23) items per study. Large variability in adherence to reporting standards was detected across individual STARD 2015 items, ranging from 0 to 100%. Nine items (1: identification as diagnostic accuracy study in title/abstract; 6: eligibility criteria; 10: index test (a) and reference standard (b) definition; 12: cut-off definitions for index test (a) and reference standard (b); 14: estimation of diagnostic accuracy measures; 21a: severity spectrum of diseased; 23: cross-tabulation of the index and reference standard results) were adequately reported in more than 90% of the studies. Conversely, 10 items (3: scientific and clinical background of the index test; 11: rationale for the reference standard; 13b: blinding of index test results; 17: analyses of variability; 18; sample size calculation; 19: study flow diagram; 20: baseline characteristics of participants; 28: registration number and registry; 29: availability of study protocol; 30: sources of funding) were adequately reported in less than 30% of the studies. Only four items showed a statistically significant improvement over time: missing data (16), baseline characteristics of participants (20), estimates of diagnostic accuracy (24) and sources of funding (30).

**Conclusions:**

Adherence to STARD 2015 among DTA studies in glaucoma research is incomplete, and only modestly increasing over time.

## Introduction

Researchers, journal editors and publishers acknowledge the need for adequate reporting of biomedical research as a means of improving the transparency and usability of journal articles [1, 2]. For this purpose, a growing set of tools has been made available to guide authors during article preparation, which have been collected in the EQUATOR framework (http://www.equator-network.org/reporting-guidelines).

The Standards for Reporting of Diagnostic Accuracy Studies (STARD) tool was released in 2003 to guide the reporting of diagnostic test accuracy (DTA) studies [3]. DTA studies are essential to investigate the performance of a new test in detecting a target disease, and can ultimately guide clinicians in the use of diagnostic tests in clinical practice [4]. An updated version of STARD has recently been published; STARD 2015 includes 9 new items compared to STARD 2003 and now consists of a list of 30 essential items that should be reported in all reports of a DTA study [5].

In the last two decades, retinal nerve fiber layer (RNFL) and optic nerve head (ONH) imaging devices for detecting glaucoma, such as optical coherence tomography (OCT), Heidelberg retinal tomography (HRT) and scanning laser polarimetry (GDx), were introduced in ophthalmic clinical practice to identify structural damages occurring early in glaucoma. However, the performance of these tests in clinical decision-making for detecting glaucoma is still debatable [6] and since their introduction, a large number of studies have been published on their diagnostic ability [7–9]. With such a large amount of evidence available, a high quality of reporting is crucial for clinicians to best appreciate the potential for bias and the internal/external validity of such studies [10]. In the case of suboptimal reporting, the available evidence could be misleading, and the potential role of these imaging tests in clinical decision-making could be misunderstood. As consequence, a biased estimate of the sensitivity/specificity of the imaging tools for detecting glaucoma could generate an over-referral of false-positive glaucoma suspects or an under-referral of false-negative glaucoma patients [11]. The application of the original version of STARD on published studies investigating the accuracy of RNFL and ONH imaging in diagnosing glaucoma showed an overall modest compliance, but they were all published in the first few years after STARD’s launch in 2003 [12–14]. More recent studies have investigated the adherence to STARD 2015 in DTA studies published in imaging journals and evaluating imaging test in different areas of interest, showing an overall moderate and variable compliance [15,16].

The aim of our study was to assess the adherence of a set of studies included in a Cochrane systematic review to STARD 2015. We investigated the overall adherence as well as for each item, whether any improvement occurred with time and which factors were associated with adherence.

STARD 2015 has been published only recently and no formal requirement of compliance with this reporting checklist has been enforced. Nonetheless, methodological knowledge underlying STARD guidance has been gradually made available over the last years [12–14]. Moreover, our study is meant to be a ‘baseline’ evaluation to guide improvement that follows STARD 2015 introduction, which could be valuable for glaucoma specialist associations in monitoring the quality of accuracy research as well as in methodological training programs.

## Methods

In this study, we considered all the 106 studies included in a Cochrane DTA systematic review published in 2016, which aimed to evaluate the diagnostic accuracy of RNFL and ONH imaging derived parameters to diagnose manifest glaucoma; details on the search and selection of studies can be found elsewhere [17].

We used the updated version of STARD to assess the quality of reporting in the included studies [5]. The STARD 2015 checklist comprises 30 items grouped in 6 domains: title and abstract, introduction, methods, results, discussion, and other information. Four STARD items (10, 12, 13 and 21) consist of two sub-items (a and b), one generally referring to the index test and the other to the reference standard.

STARD was developed to be applied to all types of diagnostic medical tests and target diseases, and some items need further specification when applied to a given test or disease. Item 10a, for example, recommends that authors report the “index test, in sufficient detail to allow replication”. Which test details are most relevant may obviously vary from test to test. In order to adapt the STARD checklist to the specific tests and target disease in the current review, we first prepared a guidance form and a data extraction form, in which specific criteria were established for scoring each STARD item. The forms were then piloted in a training session based on 5 of the included studies.

After the pilot, we drafted the final form which did not include item 2 (structured abstract), as a specific guidance for reporting abstracts has being published only recently and this should be the subject of a further study [18]. We also excluded item 13a (information available to the performers/readers of the index test) and item 25 (test-related adverse events), as they were not applicable to our index. The exclusion of item 13a was motivated by the fact that, although we know from a large body of research that knowledge of the reference standard at the time of interpreting the index test is an important source of bias [10,19], glaucoma imaging test results are always analyzed by standard, built-in software which provides an objective continuous measure of, e.g., RNFL thickness, or classifies the subject according to standard categories. Morevoer, item 25 was not considered since the test is not invasive. Overall, a total of 31 items were assessed, including several sub-items (Table 1).

**Table 1:** Compliance with STARD 2015 of the included studies with an explanation of the main patterns.

Section & Topic	N°	Item	Item reported, N (%)	Main patterns
**TITLE OR ABSTRACT**				
	**1**	Identification as a study of diagnostic accuracy using at least one measure of accuracy (such as sensitivity, specificity, predictive values, or AUC)	106 (100)	All studies used terms such as “ROC curve” and/ or “sensitivity and specificity”
**INTRODUCTION**		
	**2** **(new item)**	Structured summary of study design, methods, results, and conclusions (for specific guidance, see STARD for abstracts)	Not applicable	
	**3** **(new item)**	Scientific and clinical background, including the intended use and clinical role of the index test	10 (9.4)	Imaging features and potential use reported were frequently reported, however intended use in the clinical pathway was usually missing
	**4** **(new item)**	Study objectives and hypotheses	40 (37.4)	Study objective almost always reported, hypothesis frequently missing
**METHODS**				
Study design	**5**	Whether data collection was planned before the index test and reference standard were performed (prospective study) or after (retrospective study)	59 (55.7)	Not always clearly and explicitly reported
Participants	**6**	Eligibility criteria	103 (97.2)	Almost always reported for both cases and controls
	**7**	On what basis potentially eligible participants were identified (such as symptoms, results from previous tests, inclusion in registry)	53 (50)	We considered as properly reported when data were available for both cases and controls
	**8**	Where and when potentially eligible participants were identified (setting, location and dates)	56 (52.8)	Frequently reported the setting (where) but not the dates (when)
	**9**	Whether participants formed a consecutive, random or convenience series	44 (41.5)	We considered this adequate when information was reported only for cases
Test methods	**10a**	Index test, in sufficient detail to allow replication	104 (98.1)	Model, scanning protocol and quality criteria were considered to be reported
	**10b**	Reference standard, in sufficient detail to allow replication	106 (100)	When more than one test were used as reference standard (optic nerve head appearance and visual field), both tests must have been described in details
	**11**	Rationale for choosing the reference standard (if alternatives exist)	20 (18.9)	Positive reported when authors explained pro and cons of reference standard chosen
	**12a**	Definition of and rationale for test positivity cut-offs or result categories of the index test, distinguishing pre-specified from exploratory	97 (91.5)	Authors reported categorical data or predefined sensitivity at fixed specificity
	**12b**	Definition of and rationale for test positivity cut-offs or result categories of the reference standard, distinguishing pre-specified from exploratory	98 (92.5)	Glaucoma definition was reported for both optic nerve head and visual field reference standard
	**13a**	Whether clinical information and reference standard results were available to the performers/readers of the index test	Not applicable	
	**13b**	Whether clinical information and index test results were available to the assessors of the reference standard	29 (27.4)	Positive reported if visual field or ONH/RNFL assessor unaware of imaging test result
Analysis	**14**	Methods for estimating or comparing measures of diagnostic accuracy	105 (99.1)	ROC curve or sensitivity/specificity were almost always reported as measure of diagnostic accuracy
	**15**	How indeterminate index test or reference standard results were handled	93 (87.8)	Authors reported to have excluded index test or reference standard results as not reliable due to low quality.
	**16**	How missing data on the index test and reference standard were handled	62 (58.5)	In most cases authors reported missing data due to low quality of index test or reference standard results
	**17**	Any analyses of variability in diagnostic accuracy, distinguishing pre-specified from exploratory	27 (25.5)	Few studies reported analysis of variability in diagnostic accuracy: most cases were related to different disc size or disease severity
	**18** **(new item)**	Intended sample size and how it was determined	6 (5.7)	Not reported in almost all studies
**RESULTS**				
Participants	**19**	Flow of participants, using a diagram	0 (0)	No study reported a flow diagram
	**20**	Baseline demographic and clinical characteristics of participants	28 (26.4)	At least age, gender, intraocular pressure (IOP) and refractive status needed to be reported. Age was almost always reported, sex refraction and IOP were most often missing
	**21a**	Distribution of severity of disease in those with the target condition	105 (99.1)	Disease severity were reported both for visual field and optic nerve head as reference standard
	**21b**	Distribution of alternative diagnoses in those without the target condition	36 (34)	The reporting of IOP was considered as necessary as possible alternative diagnoses in participants without target condition
	**22**	Time interval and any clinical interventions between index test and reference standard	49 (46.2)	Time interval between index and reference standard was considered sufficient when adequately reported
Test results	**23**	Cross tabulation of the index test results (or their distribution) by the results of the reference standard	106 (100)	Also sufficient when the 2x2 table can be derived from the data available
	**24**	Estimates of diagnostic accuracy and their precision (such as 95% confidence intervals)	89 (84)	Estimates of diagnostic accuracy were almost always reported, however measures of precision were sometimes lacking
	**25**	Any adverse events from performing the index test or the reference standard		NA
**DISCUSSION**	**26** **(new item)**	Study limitations, including sources of potential bias, statistical uncertainty, and generalisability	80 (75.5)	At least one limitation was considered sufficient for a positive reporting
	**27** **(new item)**	Implications for practice, including the intended use and clinical role of the index test	34 (32.1)	Reporting of consequences of false positive and false negative test results was required
**OTHER INFORMATION**				
	**28** **(new item)**	Registration number and name of registry	2 (1.9)	Information must have been reported as explained
	**29** **(new item)**	Where the full study protocol can be accessed	2 (1.9)	Information must have been reported as explained
	**30** **(new item)**	Sources of funding and other support; role of funders	23 (21.9)	Source of funding with no details about the role of the founders was considered not sufficient for positive reporting

AUC: area under the curve; ROC: receiver operating characteristic curve; N: number of studies positively reporting the item; %: percentage with respect to the total number of included studies; ONH: optic nerve head; RNFL: retinal nerve fiber layer; (new item) indicates item newly introduced with STARD 2015 checklist.

Each study was appraised by two independent authors: one author (MM) assessed all 106 studies, while three other authors (AM, VF, GC) each independently assessed one third of the articles. For each study, each item was scored as “yes” (indicating that the item was adequately reported) or “no” (indicating that the item was not adequately reported), as explained in details in Table 2. We then assessed adherence to STARD at item level: for each item we calculated the percentage of studies scored with “yes”, as a measure of adherence to STARD. Disagreements were solved through discussion and when necessary a senior author (GV) made the final decision.

**Table 2:** Guidance followed by the raters to judge the included studies with the reasons for “yes” and “no”.

Section & Topic	No	Item	*Reasons for “yes” / “no”*
**TITLE OR ABSTRACT**			
	**1**	Identification as a study of diagnostic accuracy using at least one measure of accuracy (such as sensitivity, specificity, predictive values, or AUC)	YES: at least one measure mentioned in title or abstract, also including “diagnostic accuracy” NO: none mentioned
**ABSTRACT**			
	**2** **(new item)**	Structured summary of study design, methods, results, and conclusions (for specific guidance, see STARD for abstracts)	Not considered in this review
**INTRODUCTION**			
	**3** **(new item)**	Scientific and clinical background, including the intended use and clinical role of the index test	YES: setting in which imaging tests are used, including clinical pathway of glaucoma care explained and current test-treatment pathway summarised, potential role if the imaging test in the pathway NO: states that testing is intended to diagnose manifest glaucoma but no details given
	**4** **(new item)**	Study objectives and hypotheses	YES: both reported NO: neither or either reported
**METHODS**			
*Study design*	**5**	Whether data collection was planned before the index test and reference standard were performed (prospective study) or after (retrospective study)	YES: as explained (prospective or retrospective collection stated) NO: not explained
*Participants*	**6**	Eligibility criteria	YES: inclusion criteria reported (in case-control studies criteria for both groups have to be reported) NO: inclusion criteria not fully explained or not reported
	**7**	On what basis potentially eligible participants were identified (such as symptoms, results from previous tests, inclusion in registry)	YES: reports in what proportion patients are referred by which professionals, for which reasons (no symptom, elevated IOP, other risk factors for glaucoma), or if are self-referred. NO: no such description
	**8**	Where and when potentially eligible participants were identified (setting, location and dates)	YES: both site (clinic or hospital as a minimum) and recruitment period presented NO: neither or either reported
	**9**	Whether participants formed a consecutive, random or convenience series	YES: as explained NO: not reported
*Test methods*	**10a**	Index test, in sufficient detail to allow replication	YES: imaging test model used, protocol of acquisition used, minimum quality criteria for exclusion if any is adopted (all of these must be reported) NO: neither of either reported
	**10b**	Reference standard, in sufficient detail to allow replication	YES: visual field methods and instrument and/or optic disc assessment criteria and experience of the assessor, respectively. NO: not reported
	**11**	Rationale for choosing the reference standard (if alternatives exist)	YES: reasons for choosing either test or both. No: the rationale for choosing the reference standard not reported
	**12a**	Definition of and rationale for test positivity cut-offs or result categories of the index test, distinguishing pre-specified from exploratory	YES: cut-off reported and reference to previous research supporting it, or statement of the reasons to select it; or device pre-defined, standard positivity criteria No: not reported
	**12b**	Definition of and rationale for test positivity cut-offs or result categories of the reference standard, distinguishing pre-specified from exploratory	YES: visual field cut-off or scoring methods with reasons to identify glaucoma, clinical decision criteria for optic disc or RNFL anomaly NO: not reported
	**13a**	Whether clinical information and reference standard results were available to the performers/readers of the index test	Not considered in this review
	**13b**	Whether clinical information and index test results were available to the assessors of the reference standard	YES: reports if Visual field or ONH/RNFL imaging assessor unaware of imaging test result NO: not reported
*Analysis*	**14**	Methods for estimating or comparing measures of diagnostic accuracy	YES: general description of statistical definitions and methods NO: not reported
	**15**	How indeterminate index test or reference standard results were handled	YES: reports exclusion of low image quality results, or clarifies that they were not excluded and how they were incorporated in analyses NO: not reported
	**16**	How missing data on the index test and reference standard were handled	YES: analytic methods used to handle missing data reported for the index test and reference standard NO: not reported
	**17**	Any analyses of variability in diagnostic accuracy, distinguishing pre-specified from exploratory	YES: analyses using covariates that may have influenced accuracy and if pre-specified or post-hoc NO: not reported
	**18** **(new item)**	Intended sample size and how it was determined	YES: as explained NO: not reported
**RESULTS**			
*Participants*	**19**	Flow of participants, using a diagram	YES: as explained, including eligible, included and analysed patients NO: not reported
	**20**	Baseline demographic and clinical characteristics of participants	YES: including age, refractive status, IOP as minimum NO: not reported the minimum data required
	**21a**	Distribution of severity of disease in those with the target condition	YES: severity of glaucoma based on any classification system or as mean deviation reported NO: not reported
		Distribution of alternative diagnoses in those without the target condition	YES: reported IOP in controls groups as alternative diagnose NO: not reported
	**22**	Time interval and any clinical interventions between index test and reference standard	YES: time reported NO: not reported
*Test results*	**23**	Cross tabulation of the index test results (or their distribution) by the results of the reference standard;	YES: 2x2 data or sens/spec and n. glaucoma and no glaucoma given; or 2x2 table can be derived from existing data; mean(SD) will not be accepted for this review NO: not reported
	**24**	Estimates of diagnostic accuracy and their precision (such as 95% CI)	YES: restricted to sensitivity and specificity and 95% CI. Measure of precision (CI, SE) was sufficient for at least one measure of diagnostic accuracy. NO: neither or either reported
	**25**	Any adverse events from performing the index test or the reference standard	Not considered in this review
**DISCUSSION**			
	**26** **(new item)**	Study limitations, including sources of potential bias, statistical uncertainty, and generalisability	YES: at least comment on estimate precision, applicability of result to study question. At least one limitation was sufficient NO: no limitations were discussed at all.
	**27** **(new item)**	Implications for practice, including the intended use and clinical role of the index test	YES: at least consequences of FP and FN in the clinical pathway described
**OTHER INFORMATION**			
	**28** **(new item)**	Registration number and name of registry	YES: as explained NO: not reported
	**29** **(new item)**	Where the full study protocol can be accessed	YES: as explained NO: not reported
	**30** **(new item)**	Sources of funding and other support; role of funders	YES: both source of funding and role of funders reported. NO: neither or either reported

AUC: area under the receiver operating characteristics curve; ONH: optic nerve head; RNFL: retinal nerve fiber layer; CI: confidence interval; SE: standard error; FP: false positive; FN: false negative; (new item) indicates item newly introduced with STARD 2015 checklist.

We calculated the overall adherence to STARD 2015 as the mean number of items reported per each of the included studies. We used logistic regression to evaluate the effect of the publication year on STARD overall adherence, as well as to test whether the impact factor (IF) of the publishing journal (in the year the paper was published) could have affected the overall adherence. In the latter analysis, we formed approximate tertiles of the impact factor for 106 studies at cut-offs of 2 and 3.5, assuming that the different IFs achieved yearly by each of the 25 publishing journals were independent. The effect of the publication year on STARD adherence have been also tested for each item, separately. No adjustment for multiplicity of analyses was adopted given the exploratory nature of our study.

All calculations were made using Stata 14.2 software (StataCorp, College Station, TX).

## Results

### Characteristics of included studies

Readers can refer to Michelessi et al for details on the included studies [17]. In short, we included 106 studies, of which 40 studies (5574 patients) assessed the diagnostic accuracy of GDx, 18 studies (3550 patients) that of HRT, and 63 (9390 patients) that of OCT. Twelve of these studies compared two or three tests. Sixty-seven studies used visual field (VF) damage plus ONH glaucomatous optic neuropathy as the reference standard; the remaining 37 studies relied on either VF damage only (29 studies) or ONH/RNFL damage only (10 studies) as definition criteria for confirming glaucoma. Studies were published between 2003 and 2014; median impact factor was 2.6 (interquartile interval 1.0 to 3.7).

### Adherence to STARD 2015

Overall adherence was 54.1% for 3,286 individual rating across 31 items, with a mean of 16.8 (SD: 3.1; range 8–23) items per study. Table 1 presents the adherence to STARD 2015 for each item with an explanation of the main patterns.

Overall, a large variability in adherence to reporting standards was detected across STARD 2015 items, ranging from 0 to 100%. Nine items were adequately reported in more than 90% of the studies: identification as a study of diagnostic accuracy in the title (item 1); eligibility criteria (item 6); index test (item 10a) and reference standard (item 10b) in sufficient detail to allow replication; definitions of test positivity cut-offs for the index test (item 12a) and reference standard (item 12b); methods for estimating measures of diagnostic accuracy (item 14); severity spectrum of diseased (21a); cross-tabulation of the index test and reference standard results (item 23). Specifically, three items were reported in all the included studies (items 1, 10b and 23).

Conversely, 10 items showed adherence to STARD in less than 30% of the studies: scientific and clinical background, including the intended use and clinical role of the index test (item 3); rationale for the reference standard (item 11); whether assessors of the reference standard were blinded (13b); analyses of estimate variability (item 17); sample size calculation (item 18); study flow diagram (item 19); baseline characteristics of participants (item 20); registration number and registry (item 28); availability of study protocol (item 29); sources of funding (item 30).

Four items showed mixed reporting among the included studies, with adherence close to 50% of reporting: study design as prospective or retrospective (item 5); setting, location and dates (item 8); basis for identifying potential eligible participants (item 7); time interval and intervention between index test and reference standard (item 22).

### Trends in and association with adherence

Overall, a modest increase of adherence was found with publication year (OR: 1.03 per year, 95%CI 1.00 to 1.05; p = 0.032).

S1 Fig shows the fraction of adherence to STARD 2015 for each item throughout the period encompassing the publication dates of all included studies, comprising 36 studies published between 2003 and 2009 and 70 studies published between 2010 and 2014.

While most trends were towards an improvement of adherence over time, a statistically significant improvement in reporting was found for only four items (OR: odds ratio of adherence per one year): how missing data on the index test and reference standard were handled (item 16, OR 1.22, p = 0.003); baseline demographic and clinical characteristics of participants (item 20, OR 1.24, p = 0.010); estimates of diagnostic accuracy and their precision (such as 95% confidence intervals) (item 24, OR 1.22, p = 0.018); sources of funding and other support; role of funders (item 30, OR 1.20, p = 0.037). No item showed a significant decrease in adherence over time.

The journals publishing the largest number of studies were Investigative Ophthalmology and Vision Science (n = 19), Ophthalmology and Journal of Glaucoma (n = 16), and the American Journal of Ophthalmology (n = 12). We found slightly better overall adherence for journals with IF 3.5 or more versus less than 2 (OR: 1.22, 95%CI 1.02 to 1.47; p = 0.033).

A mixed-effect linear model showed that most of the variance was found at the item level, while variance at the journal level was more than 100 times smaller, suggesting little effect of a journal on adherence.

### Patterns of adherence and non- adherence

All included studies were identified as a diagnostic accuracy study in the title or abstract, mainly by reporting measures of accuracy such as ROC curve, sensitivity or specificity (item 1).

Only 9% of the studies were considered to have reported the scientific and clinical background adequately (item 3); although the authors often reported the imaging test characteristics and its ability to detect damage, the intended use and clinical role of the index test along the diagnostic pathway were lacking in most cases. Study objectives and hypotheses (item 4) was poorly reported (38% of cases): although objectives were almost always reported, the scientific hypothesis was often missing. Items related to study design and participant enrollment were variably reported across included studies. With the exception of eligibility criteria (item 6), which was reported in 97% of the studies, the other items were adequately reported in only about half of the studies: the prospective or retrospective nature of the study (item 5, 55% of the studies), the basis on which eligible potential participants were identified (item 7, 49% of the studies), whether participants formed a consecutive, random or convenient series (item 9, 42% of the studies). Setting location and dates (item 8) were reported in 53% of the studies, with dates more often missing.

Imaging test devices and reference standard used (items 10a and 10b) were clearly reported in 98% and 100% of the studies, respectively. The definition of and rationale for test positivity cut-offs (items 12a and 12b) were properly reported both for the index test and reference standard in 92% and 98% of the studies, respectively. On the contrary, authors reported the rationale for choosing the reference standard (or the existence of an alternative) only in 20% of the studies, and information about masking of assessors of the reference standard was reported in 27% of the studies.

The methods for estimating or comparing measures of diagnostic accuracy were reported in almost all studies (99%). How indeterminate results were handled (item 16) was reported in 88% of the studies; the exclusion of low quality scans was the main method for handling indeterminate results. On the contrary, how missing data were dealt with (item 17) was reported in only 58% of cases. The authors rarely specified how missing data were dealt with, and in most cases missing data could only be computed by comparing the number of enrolled patients with those included in the final analysis.

Analyses of variability in diagnostic accuracy were reported in only 27% of cases (in most cases related to the disc size of the ONH or disease severity of participants), and only 6% of the studies reported the intended sample size and how it was determined.

All studies reported a cross-tabulation of the results of the index test with the results of the reference standard, or data to derive this cross-tabulation (item 23), and 99% of the studies reported the distribution of disease severity in participants with the target condition (item 21b). Most studies (84%) reported estimates of diagnostic accuracy and their precision (item 24). When this item was not properly reported (16%), a measure of precision such as 95% confidence intervals was missing.

Baseline demographic and clinical characteristics of participants (item 20) was considered properly reported if at least age, gender, intraocular pressure (IOP) and refractive status were reported, which was the case in 26% of the studies. Age was almost always reported, while sex, refraction and IOP were most often missing. No study presented a flow diagram of participants (item 19).

Study limitations (item 26) were reported in 75% of the studies. The case-control design and a low generalizability due to the characteristics of included participants (such as disease severity or ethnicity) were mainly reported as limitations. Only 32% of the studies reported implications for practice, and for the intended use and clinical role of the index test (item 27), sometimes referring to changes between pre- and post-test probability.

Sources of funding, including the role of funders (item 30), were reported only in 21% of the studies; frequently, authors did report the source of funding but did not describe the funders’ role. Registration number and name of registry (item 28) as well as full study protocol details (item 29) were reported in only 2% of the studies.

## Discussion

Our review investigated adherence to STARD 2015 in a large set of DTA studies evaluating the diagnostic performance of imaging devices for detecting manifest glaucoma. In general, the completeness of reporting was modest and highly variable across items.

Overall, a mean of 16.8 out of 31 items, ranging from 8 to 23 items, were adequately reported for the 106 studies included.

Across the 31 items assessed in our review, some items showed an almost perfect adherence to STARD 2015 but the reporting of other items was definitely very poor. Items with the lower level of reporting included the scientific and clinical background (item 3), the basis on which eligible potential participants were identified (item 7), and the setting location and dates (item 8). This information is crucial, as the performance of a test is not fixed, but may vary if applied in different settings and among patients with different characteristics [2]. The lack of this information makes it difficult to evaluate the generalizability of the results. Moreover, only one third of the studies discussed the consequences of false positive and false negative results in the clinical pathway. This could increase the risk of a misunderstanding how the test could change the post-test probability of disease.

Poor reporting was also found regarding the rationale for choosing the reference standard (item 11), masking of assessors of the reference standard (item 13b), and handling of missing data (item 16). The use of different reference standards can introduce heterogeneity in test accuracy, as one reference standard may be more accurate than the other. Review bias can arise when the index test results are known to the assessor of the reference standard. Improper handling of missing data can also be associated with biased results.

Time interval between index test and reference standard (item 22) was reported in half of the studies. Glaucoma is a progressive disease and functional/structural damage may occur over time not concurrently [20]. Different time intervals between structural index tests and functional reference standards may affect the estimated diagnostic accuracy.

Demographics and clinical characteristics of participants (item 20) and alternative diagnoses in those without the target condition (item 21b) were also often inadequately reported. Details on the population enrolled permit judgement of the potential for selection and spectrum bias and decide on the applicability of the results to other populations.

One item was never reported in the included studies: participant flow using a diagram. STARD 2015 strongly recommends the use of a flow chart to facilitate the reader’s comprehension of study design and the flow of participants along the study process [21].

Other studies have evaluated the adherence to STARD 2015 in DTA studies. Hong et al. investigated 142 DTA studies published in imaging journals, and found the mean number of reported STARD items was 16.6/30 with an overall adherence of 55%, which is similar to our results with the updated tool [15]. A better adherence to STARD was found by Choi et al., who investigated 63 DTA studies published between 2011 and 2015 in a single specialty journals (Korean Journal of Radiology) with a mean adherence of 20/27 items (74%) [16]. We acknowledge that adherence could vary according to type of diagnostic test (imaging, biochemistry, histopathology), as well as specialty. Moreover, the specific guidance adopted by different reviewers to score STARD adherence might introduce differences. Despite these potential sources of variability, the limited number of studies which have been conducted on adherence to STARD 2015 suggest there is room for improvement.

We also found the overall completeness of reporting slightly improved over the years. Only 4 items (13%) showed a significant improvement over time but, despite this improvement, 3 of these items were only reported in less than 60% of cases. Korevaar et al. identified 16 surveys analyzing the reporting of 1496 DTA studies, and found moderate improvement of reporting in the first years after STARD’s introduction, but with substantial heterogeneity among studies [22]. In 2015, Fidalgo et al. investigated the use of STARD 2003 in 58 studies on automated perimetry for glaucoma and recorded suboptimal reporting with no improvement between 1993–2004 and 2004–2013 [23].

We also hypothesized that journal IF could have affected the completeness of reporting. Overall, a higher IF was associated with only slightly better reporting, suggesting that the need for improved reporting involves both journals with low and high IF.

The Cochrane review from which our studies were retrieved [17] assessed the methodological quality of the studies using the QUADAS-2 tool [19]. We found the relationship between adherence to STARD 2015 and methodological quality with QUADAS 2 was only partial, which is the subject of a different methodological study (accepted).

The general picture emerging from the literature is that the completeness of reporting of imaging studies in different disciplines is only moderate and DTA studies of imaging test for detecting glaucoma are in line with these findings. The STARD group members and promoters encouraged journal editors to prescribe the use of their checklist in submissions. Although this led to some improvement of overall adherence to STARD, many items were still not reported in studies published in journal adopting the STARD checklist [15].

Our review has limitations and strength. All the included studies were published before STARD 2015 was introduced, so that authors were only able to use the previous version of STARD, which was published in 2003. However, we included a very large set of studies (70% of which were published after 2010) and each study was judged by two independent reviewers to improve the reliability of the assessment. Another limitation is that we evaluated only a specific disease entity-index test(s), which may limit generalizability. Moreover, we used a cohort of studies that met inclusion into a Cochrane review which, depending on the inclusion criteria applied, may have biased the included studies to be of higher ‘quality’ or better reported than those that might not have met inclusion for the review.

Our study offers an updated focus on the completeness of reporting of DTA studies in ophthalmology, specifically in glaucoma research. Our study also confirms that the adherence of glaucoma imaging DTA studies to STARD 2015 is modest and that more work and effort is needed to improve the completeness of research. Finally, this study has also set the basis for future evaluations of how the introduction of STARD 2015 will change the reporting of DTA studies on glaucoma over the next few years.

## Supporting information

S1 Fig Fraction of adherence to STARD 2015 throughout the period encompassing the publication dates of all included studies (2003–2014). Each item is numbered from 1 to 30.
(TIF)

S1 File Dataset collected and used for the analysis. (XLSX)
