# Supplementary figures and images for: Diagnostic accuracy research in glaucoma is still incompletely reported: An application of Standards for Reporting of Diagnostic Accuracy Studies (STARD) 2015
Source: PLoS One. 2017 Dec 14;12(12):e0189716. doi: 10.1371/journal.pone.0189716 (PMC5730182; doi:10.1371/journal.pone.0189716)

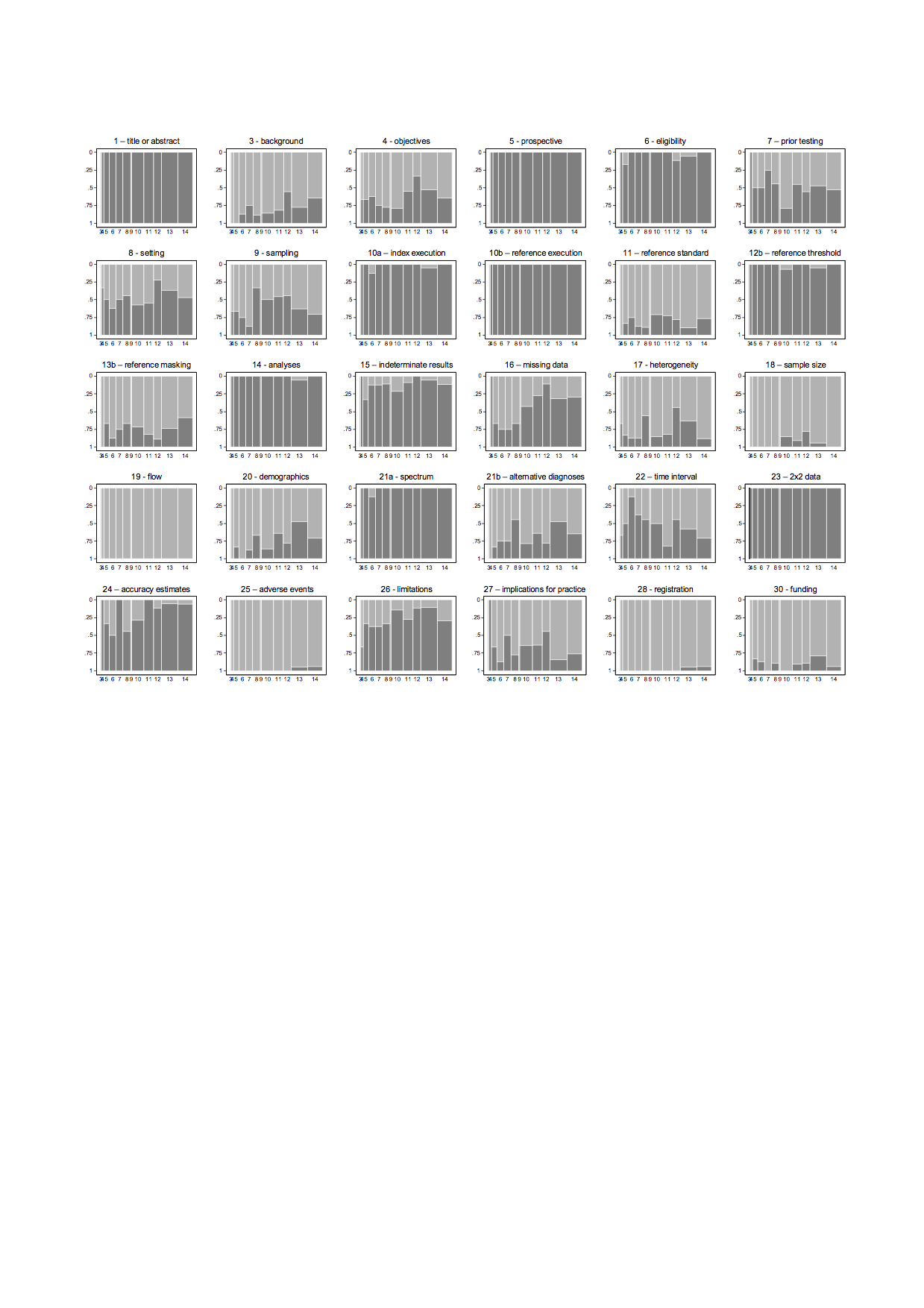

Supplement: S1 Fig — Each item is numbered from 1 to 30. (TIF) [file pone.0189716.s001.tif]
